# Management of Ankyloglossia in a Six-Year-Old Child After Cleft Lip and Palate Surgery: A Case Report

**DOI:** 10.7759/cureus.31108

**Published:** 2022-11-04

**Authors:** Sakshi Kabra, Nilima R Thosar, Monika Khubchandani

**Affiliations:** 1 Pediatric and Preventive Dentistry, Sharad Pawar Dental College and Hospital, Datta Meghe Institute of Medical Sciences, Wardha, IND

**Keywords:** local anesthesia, speech therapy, cleft lip and palate, diode laser, ankyloglossia

## Abstract

Ankyloglossia or tongue-tie is caused by an excessively short, thick lingual frenum that restricts the normal movements and functions of the tongue. It has a higher prevalence in infants than in children and adults. In the present case, a six-year-old male came with his parents with a chief complaint of difficulty in speech. His medical history revealed that he had a congenital cleft lip and cleft palate, for which he had undergone surgery soon after his birth. He was categorized by Kotlow classification as Class II (moderate ankyloglossia). Under local anaesthesia, diode laser surgery was planned to treat the tongue-tie. The patient showed excellent healing after a one-week follow-up. An increase in tongue movements was seen and the patient was put in consultation with a speech therapist.

## Introduction

Tongue-tie, also known as ankyloglossia, is a congenital anomaly marked by a small lingual frenulum that may limit tongue movement and have an adverse effect on function [1]. Ankyloglossia derives from the Greek words "agkilos" (curved) and "glossa" (tongue). Ankyloglossia is the tongue tip's inability to protrude because of a small lingual frenulum [2].

The tongue muscle is the only organ in the body with one end attached and the other being free [3]. Ankyloglossia affects 0.1% to 10.7% of the population. According to reports, newborns have a higher prevalence (1.72%-10.7%) than kids, teenagers, or adults (0.1%-2.08%) [4]. A male-to-female ratio of ankyloglossia is 2.5:1. The anomaly appears to be more prevalent in males for unspecified reasons [5]. Some rare syndromes, such as van der Woude syndrome, Opitz syndrome, and X-linked cleft palate syndrome, are associated with tongue-tie [3].

According to Kotlow’s classification of ankyloglossia [6], it is noted that ankyloglossia can be one of the following four types depending on the clinically visible free tongue: Class I - Mild ankyloglossia with 12-16 mm clinically visible free tongue. Class II - Moderate ankyloglossia with 8-11 mm free tongue. Class III - Severe ankyloglossia with 3-7 mm free tongue. Class IV - Complete ankyloglossia.

## Case presentation

**Figure 1:**
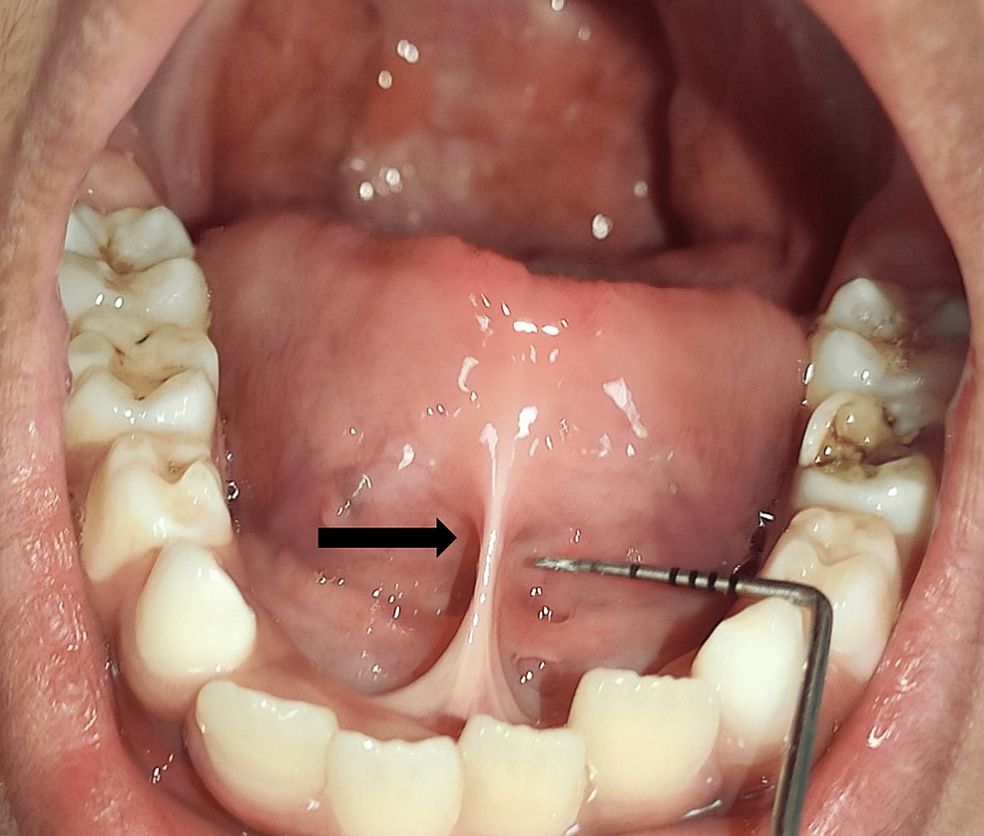
Limited upward extension of tongue

A six-year-old child reported to the department of pediatric and preventive dentistry with the chief complaint of difficulty in speech and pronunciation. His medical history revealed that he had congenital cleft lip and cleft palate, for which he underwent surgery immediately after birth. On intraoral examination, the patient had a secondary fistula formation on the palate, and a thick inferior lingual frenum attached 8 mm from the tip of the tongue (Figure 1).

There were noticed restricted tongue motions, including protrusion, lateral movements, and an inability to touch the palate with the tongue tip. The child was categorised by Kotlow's classification as having Class II or moderate ankyloglossia.

**Figure 2:**
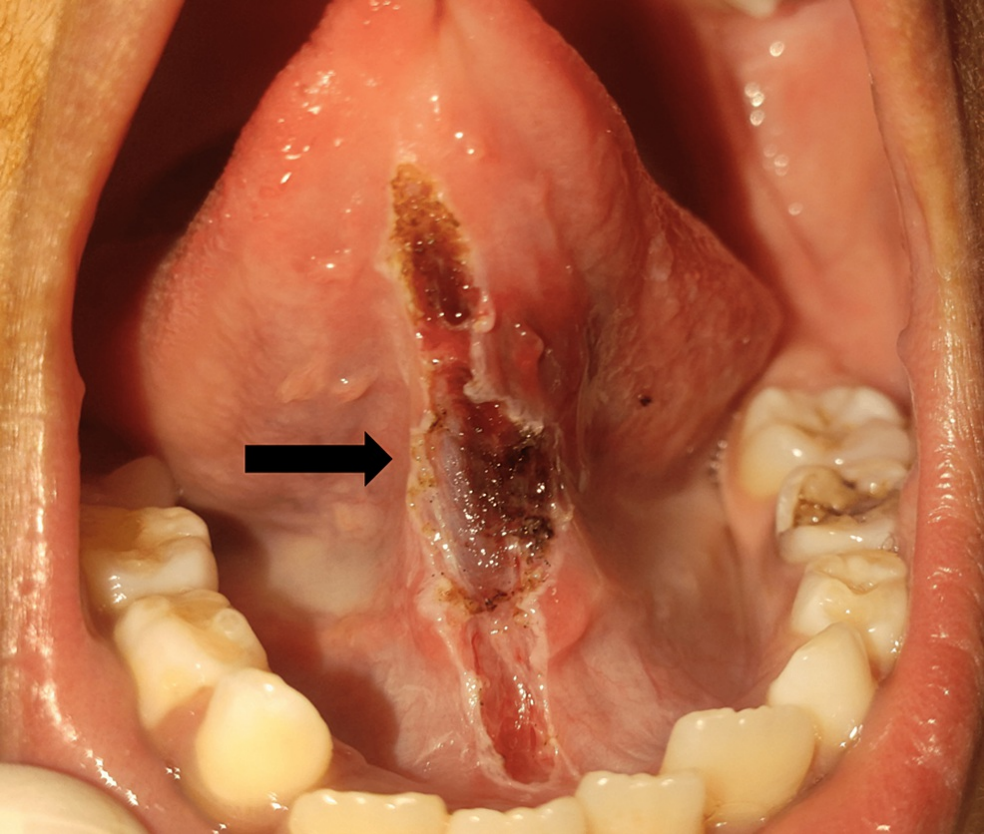
Incision of lingual frenum done

A lingual frenectomy was planned utilising a diode laser set to 980 nm in continuous mode at 1.8 Watts (W). For local anaesthetic, a cartridge containing 2% lidocaine with 1:80,000 epinephrine was used. The operator and the child were given the proper eye protection and given the required care. The lingual frenum was dissected after the tongue tip had been immobilised. The densely embedded muscle fibres were detached from the floor of the mouth in order to permit enough and appropriate tongue movement (Figure 2).

**Figure 3:**
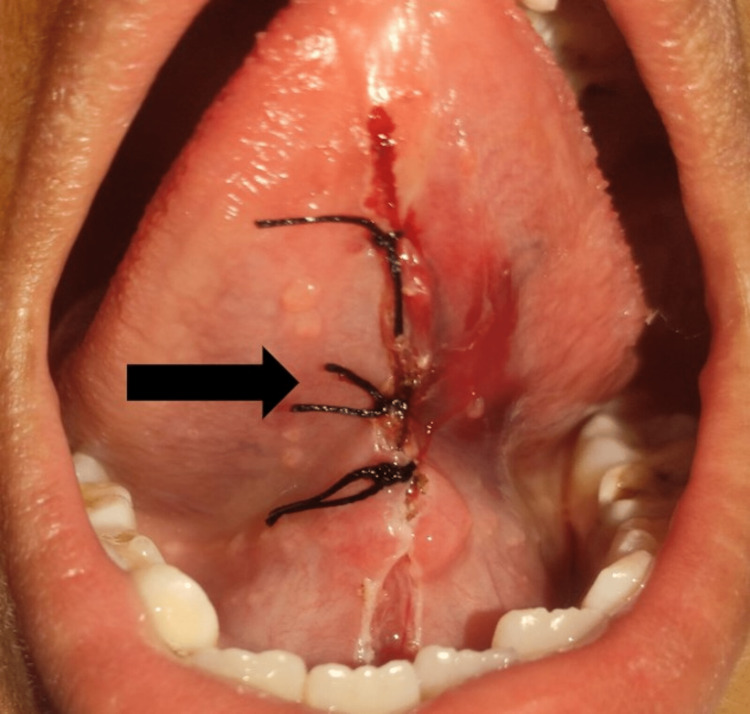
Immediate post-operative view revealed free movement of tongue

**Figure 4:**
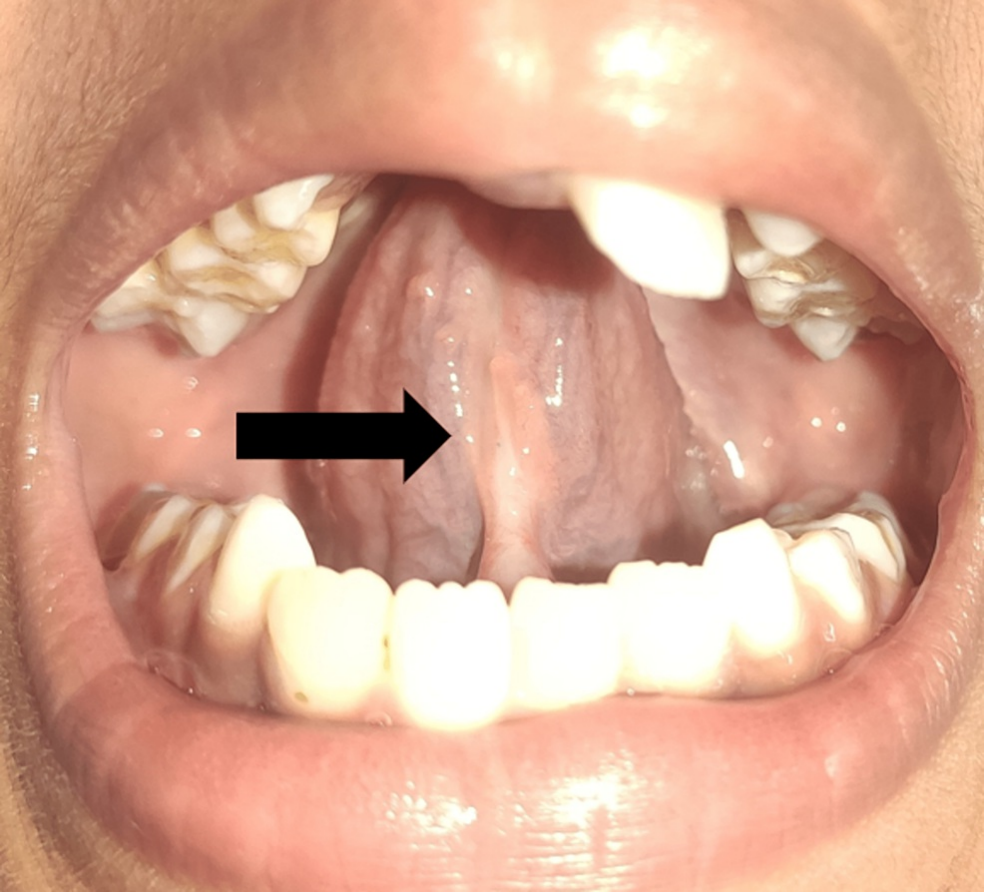
One-week post-operative view showed complete healing of tissues

After surgery, sutures were given (Figure 3), and the child was called again after one week. There was no delayed bleeding, and the site healed satisfactorily after one week (Figure 4).

## Discussion

Specific speech impairments are caused in some children by ankyloglossia, whether partial or complete. The interference with articulation is consistent with our scenario even though it neither delays nor prevents the commencement of speaking [7]. Some tongue sounds, such as "t", "d", "l", "th", and "s", are difficult to pronounce. Improper chewing and swallowing of food in children with tongue-tie can increase the risk of gastric distress and bloating, bedwetting, and snoring while they sleep. [8].

According to Lamba et al. [9], management of ankyloglossia is dependent on the location, the severity of tongue mobility restriction, and associated functional restrictions in addition to the patient's age. Kara et al. [10] and Adelaimi and Mahmood [11] reported that using a surgical laser instead of a scalpel or blade is a great and reliable choice for pediatric frenectomies, Therefore in the present case, instead of a scalpel or blade, diode laser was preferred for the surgery.

Diode lasers have an easy-to-use beam delivery technology that uses an optical flexible fibre handpiece. Laser is strongly absorbed by haemoglobin and poorly absorbed by water. Diode lasers can effectively seal the capillaries by causing protein denaturation and activating the synthesis of clotting factor VII (stable factor). Additionally, these lasers have antimicrobial qualities and are recommended for oral soft-tissue procedures with minimum bleeding close to tooth structures. Water cooling is essential since they may quickly raise the temperature if they are applied for an extended period of time to irradiated tissue [12]. In our case, laser surgery was chosen to cut the tissue and preserve as much as possible to prevent scarring of the tongue. Laser leads to faster and safe healing. It also helps to minimize postoperative bleeding.

In cases of cleft palate with ankyloglossia (CPX) patients, TBX22 gene (T-box transcription factor) expression is evident in the palatal shelves, where it is prominent before being elevated to a horizontal position over the tongue. Additionally, TBX22 mRNA was found near the frenulum at the base of the tongue, which is the location where ankyloglossia is present in CPX patients [13]. in the present case, there was a history of cleft lip and palate with ankyloglossia, which may be associated with TBX22 gene. After surgical correction of tongue-tie, the patient was referred for speech articulation to a speech therapist.

## Conclusions

Based on the degree of lingual adhesion, ankyloglossia that could lead to oral issues can be easily corrected via frenectomy. Many kids with ankyloglossia might not express their discomfort with the difficulties it causes. Therefore, it is crucial to provide guidance to the patient or parent so that newborns and children can receive the proper care at the appropriate time. In comparison to conventional scalpel/blade methods, lingual frenectomy as a management therapy for ankyloglossia, via laser surgery, offers a better outcome for both the young patient and the pediatric dental professional. However, following surgical repair, the patient should be directed to a speech therapist. 
